# Determinants of bed net use in children under five and household bed net ownership on Bioko Island, Equatorial Guinea

**DOI:** 10.1186/1475-2875-10-179

**Published:** 2011-06-29

**Authors:** Alberto L García-Basteiro, Christopher Schwabe, Cynthia Aragon, Giovanna Baltazar, Andrea M Rehman, Abrahan Matias, Gloria Nseng, Immo Kleinschmidt

**Affiliations:** 1 Preventative Medicine and Epidemiology Unit, Hospital Clínic, C/Villarroel 174, CP 08036, Barcelona, Spain; 2 Barcelona Centre for International Health Research (CRESIB, Hospital Clínic-Universitat de Barcelona), Barcelona, Spain; 3 Medical Care Development International, 8401 Colesville Rd., Suite 425, Silver Spring, MD 20910, USA; 4 Medical Care Development International, Malabo, Equatorial Guinea; 5 MRC Tropical Epidemiology Group, London School of Hygiene and Tropical Medicine, Keppel Street, London WC1E 7HT, UK; 6 Ministry of Health and Social Welfare, Malabo, Equatorial Guinea

## Abstract

**Background:**

As part of comprehensive malaria control strategies, the Bioko Island Malaria Control Project (BIMCP) distributed 110,000 long-lasting insecticide-treated nets (LLIN) in late 2007 with the aim of providing one net for each sleeping area. Despite attaining initially very high levels of net coverage and net use, many children under five years of age did not sleep under a net by 2009, according to annual malaria indicator surveys. The aim of this study was to assess the determinants of bed net use in children under five and bed net ownership of the households in which they live.

**Methods:**

Using data from annual cross-sectional household surveys of 2008 and 2009, we investigated factors associated with sleeping under a mosquito net the night prior to the survey, and a households owning at least one net, in all households which had at least one child under five years. Amongst others, caregiver's knowledge of malaria and household characteristics including a socio-economic score (SES), based on ownership of household assets, were analysed for their effect on net ownership and use.

**Results:**

There was a decline of around 32% in the proportion of households that owned at least one net between 2008 and 2009. Higher household bed net ownership was associated with knowing how malaria was prevented and transmitted, having the house sprayed in the previous 12 months, having fewer children under five in the household, and children being sick at some point in the previous 14 days. Higher bed net use in children < 5 was associated with being sick at some point in the last 14 days prior to the survey, living in an urban area, more years of education of the head of the household, household ownership of at least one ITN (as opposed to an untreated net) and the year in which the survey took place.

**Conclusions:**

The big fall in bed net use from 2008 to 2009 was attributable to the striking decline in ownership. Although ownership was similar in rural and urban areas, rural households were less likely to protect their children with bed nets. Knowledge about malaria was an important determinant of bed net ownership. Further research is needed to elucidate the decline in bed net ownership between 2008 and 2009.

## Background

Children under five years of age, together with pregnant women, have been identified as the most vulnerable risk group for malaria, with 88% of all deaths in sub-Saharan Africa attributed to malaria occurring in children under-five [1]. In Equatorial Guinea, malaria is a major endemic disease, responsible for about 28% of the deaths in children under five in 2008 [2]. Between 2004 and 2008 there has been a drastic reduction in the prevalence of infection with malarial parasites from 42% to 22% in children between two and five years of age in Bioko, Equatorial Guinea's main island, following a comprehensive malaria control intervention. This was associated with a two-thirds reduction in under-five mortality (it fell from 152 to 55 per 1,000 births) [3].

Insecticide-treated nets (ITN) have been widely shown to be effective in reducing childhood morbidity and mortality through reducing mosquito bites while sleeping [4,5]. ITNs have been shown to be the most cost-effective measure to reduce malaria transmission [6,7]. However, the target of 80% coverage of children sleeping under a net by 2010 set by RBM (Roll Back Malaria) Programme [6] is ambitious for most countries. According to the World Malaria Report (WMR) 2009 [1], 31% of African households owned an ITN in 2008, and 24% of children slept under an ITN (WMR compiles information from a subset of African countries)[1]. In Bioko, following the mass delivery of bed nets in late 2007, over 95% of households owned at least one ITN, and over 70% of children from two to five slept under an ITN [3].

As has been previously reported, even when there are bed nets available in the household, children under five, the most vulnerable group, do not always have access to them [8-10]. Although previous studies have investigated the determinants of bed net utilization and ownership [8,11-17], the results remain inconclusive.

In a study conducted in five African countries [18], bed net use was associated with age. Among under-fives, there was a clear decline in use as the child grew older. The same decline in use is shown in children whose mothers sleep under a mosquito net in Uganda [19]. Low use in children under five compared to individuals of other age groups was also found in the Western Kenya [20]. Being male was directly and inversely associated with bed net use depending on the setting [11,14,18,21]. Some studies found higher socio-economic status SES related to higher ownership and utilization [16], while others did not find such an association [13,17,22].

Variables such as occupation and education levels of the household appeared to be directly associated with bed net use in some studies [14,16]. In Ethiopia it was found that the conical shape of the bed net or having to pay for the bed net (instead of getting it for free) was associated with higher proportions of people sleeping under a net [12]. Other variables which were associated positively with using a net were: living in an urban area compared to rural area [23], and sleeping with the mother [19].

The Bioko Island Malaria Control Project (BIMCP), which is funded by a consortium led by Marathon Oil Corporation and the Government of Equatorial Guinea, has conducted intensive malaria control activities in Bioko since 2004. These included free semi-annual rounds of IRS since 2004 targeting all households on the island, introduction of free artemisinin-based combination therapy (ACT) for pregnant women and children under 15 in 2005 (subsequently extended free-of-charge to the entire population in 2010), free Intermittent Preventative Treatment of pregnant women (IPTp) in 2005 as well as extensive information, education and communication (IEC) campaigns. The latter used mass media (radio, television, and printed materials) as well as community-based interpersonal communications techniques. In 2007, the BIMCP distributed around 110,000 long-lasting insecticidal nets (LLIN) on Bioko Island free of charge. This intervention consisted of a door-to-door campaign with the intention to cover all sleeping areas in all households on the island. Householders were assisted with hanging nets where necessary. The mass distribution campaign resulted in very high levels of net ownership and use, but theses declined rapidly over time [3]. All interventions targeted the entire population of Bioko equally.

The objective of this study was to identify factors associated with use of bed nets in children under five in Bioko. As a secondary objective, this study aimed to identify the particular characteristics of the households that own bed nets.

## Methods

**Figure 1:**
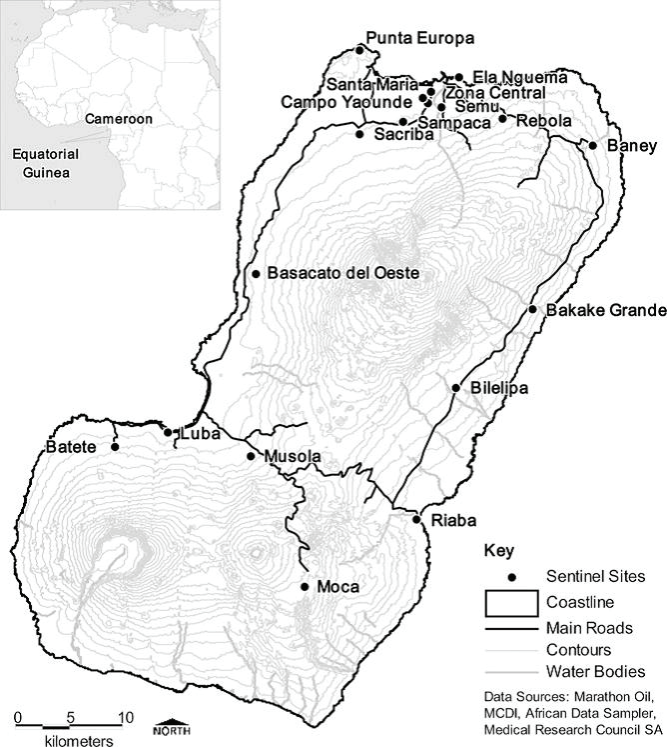
**Sentinel sites in Bioko Island participating in the study**. Map reproduced from Kleinschmidt *et al *[26]. MCDI = Medical Care Development International; SA = South Africa.

Data from two post-ITN mass distribution cross sectional annual surveys conducted by the BIMCP during February to April in 2008 and from August to September in 2009 respectively were analysed. The survey questionnaires were adapted from the Roll Back Malaria Monitoring and Evaluation Reference Group Malaria Indicator Survey and conducted in 18 sentinel sites across the island, as described elsewhere in more detail [24] (Figure 1) [25,26].

Households were sampled randomly from household census lists at each sentinel site. The sentinel sites include about 95% of houses in Bioko (45000-50.000 houses) according to a household census conducted by BIMCP (unpublished data, BIMCP). The surveys are, therefore, broadly representative of the entire population of Bioko of around 260,000 inhabitants [27].

The original sample size of the surveys was calculated so that a change in prevalence of infection of 20% could be detected compared to the previous year at the same site with 80% power and a 5% type of 1 error level [28]. In subsequent years the sample size was increased to be able to detect smaller changes in prevalence. For this study, only data from households with children under five years of age were analysed.

The outcome variables were "*having slept under any net the night before the survey*" for each child under five, and "*owning at least one bed net*" for each household with a child under five. Most explanatory variables were collected at the household level, from the responses given by the person interviewed in the household, usually the head of household or caregiver. Data on knowledge of the person interviewed in the household was obtained through a series of questions concerning malaria prevention, malaria symptoms and malaria treatment (As an example: there was the question: *How is malaria transmitted? *There were seven different options, including the correct answer "*mosquitoes"*). For the purpose of analysis, these questions were converted into three binary variables: "*Is the use of bed nets an effective way to prevent malaria*?", "*Can mosquitoes transmit malaria?*" and "*Is fever a symptom of malaria*?", each with a *yes *or *no *value.

Other explanatory variables for the analysis of bed net use were: age and gender of the child, whether the house was sprayed with IRS in the last 12 months, disease status of each child (under-five) in the last 14 days (according to household interviewee), socio-economic status, age and education of head of household, type of eaves (open or closed), setting (rural or urban), existence of at least one ITN in the household. In this analysis only data on children under five living in households with at least one bed net (since a necessary condition to use a bed net is to own it). This dataset contained information relating to the individual child and data relating the household they lived in.

Apart from the knowledge questions, the analysis on bed net ownership included the following explanatory variables: age and gender of the child, whether the house was sprayed with IRS in the last 12 months, disease status of each child (under-five) in the last 14 days (according to household interviewee), socio economic status, age, sex and education of head of household, setting (rural or urban) knowledge of availability of ACT for free, household size, living in the house for the last 12 months or more, and whether any woman in the household had been pregnant in the last year. In this analysis, data relating to households with children under-five were used (since bed nets are owned by households).

Socio-economic status was calculated using Principal Component Analysis (PCA), which generates a single weighted index combining asset ownership, household infrastructures and basic amenities like sanitation, following the recommendations of Vyas *et al *[29]. Variables with very low frequencies (< 1%), were excluded due to their low capacity for differentiating households from each other. The SES index was divided into quintiles.

Multiple logistic regression models were built to assess the effect of each explanatory variable on the outcome variable, adjusting for confounding. All the variables that showed at least some evidence of association (p-value < 0.1 in the univariate analysis) were tested in the multivariable analysis. The regression models were built with a forward step-wise strategy. The variables with lowest p-value obtained in the univariate analysis were tested first. Statistical analysis has been conducted using STATA v.11.

Ethics approval for the original surveys was granted by the Equatorial Guinea Ministry of Health and Social Welfare and London School of Hygiene and Tropical Medicine (approval number 556).

## Results

A total of 5,151 children under five from 3,210 households (average of 1.6 children per household) with children in this age group were included in the 2008 and 2009 surveys.

Twenty-eight variables were included in the PCA analysis (Table 1) The SES score had a mean of 0 and ranged from -5.08 to 8.15. Ninety seven percent of the households analysed in 2008 owned at least one bed net and 81.2% owned at least one ITN. In 2009, 65% of the households owned at least one ITN and 47% owned at least one ITN. Table 2 shows the most important variables describing household characteristics per survey year.

**Table 1:** Results from the Principal Component Analysis (PCA) Mean, Standard Deviation (SD) and Factor Score for the variables included in the First Principal Component (n = 3210)

Variable description	Mean(SD)	Factor Score (1st Component)
**Assets**		
Television	0.759(0.428)	0.291
Bicycle	0.045(0.206)	0.108
Motorcycle	0.014(0.117)	0.058
Motorboat	0.014(0.116)	0.049
Radio	0.666(0.472)	0.199
Refrigerator	0.612(0.487)	0.302
Car	0.161(0.367)	0.215
Telephone	0.874(0.332)	0.228
Rooms	4.697(2.347)	0.175

**Source of lighting**		
Private generator	0.354(0.478)	0.079
Public electricity	0.574(0.494)	-0.003
Gas or petrol lamp	0.045(0.207)	-0.135

**Source of water supply**		
Piped water from public tap	0.520(0.499)	-0.076
River/Stream/Manantial	0.314(0.464)	0.003
Protected well	0.052(0.222)	0.048

**Type of floor**		
Cement	0.702(0.458)	-0.210
Stone	0.225(0.418)	0.320
Dust	0.065(0.246)	-0.151

**Sanitation facilities**		
Flush toilet	0.310(0.462)	0.229
Traditional latrine	0.267(0.443)	-0.206
Ventilated latrine	0.252(0.434)	0.027

**Type of walls**		
Cement	0.363(0.481)	0.248
Wood	0.616(0.486)	-0.226

**Source of cooking fuel**		
Gas	0.256(0.437)	0.284
Kerosene	0.545(0.498)	-0.071
Wood	0.173(0.378)	-0.223

**Other**		
Not open Eaves	0.627(0.484)	0.250
Window Glass	0.050(0.219)	0.173

**Table 2:** Characteristics of the households included in the study (years 2008 and 2009)

	2008	2009
	n (%)	n (%)
**Households surveyed**	1517(47.26)	1693(52.74)
**Households owning at least one net**		
Yes	1380 (96.6)	1073(64.8)
No	49(3.4)	582(35.2)
**Household owning at least one ITN**		
Yes	1169(81.8)	774(46.8)
No	260 (18.2)	881(53.2)
**Household socioeconomic status**		
*lowest*	360(54.9)	296(45.12)
*2nd quintile*	327(49.8)	330(50.23)
*3rd quintile*	314(49.37)	322(50.63)
*4rd quintile*	283(45.65)	337(54.35)
*highest*	233(36.35)	408(63.65)
**Area**		
*urban*	557 (36.3)	979(63.7)
*rural*	960 (57.4)	714(42.7)
**Education of head of household**		
*none*	40(25.0)	120(75.0)
*1-4*	178(66.4)	90(33.6)
*5-9*	895(50.9)	865(59.2)
*> 9*	366(40.2)	544(59.8)

**Household size **(mean and SD^1^)	5.7(2.5)	5.6 (2.3)
**Age members of household **(mean and SD^1^)	20.1 (8.6)	17.2(5.7)
**Rooms per household **(mean and SD^1^)	4.3 (2.0)	4.4 (1.8)
**Beds per household **(mean and SD^1^)	2.8(1.5)	2.4 (1.2)
**Age head of household **(mean and SD^1^)	39.1(17.5)	39.2(16.3)

^1^SD = Standard Deviation.

The mean number of individuals per household who slept in houses that had at least one net or ITN was 3.5 (SD 3.6) and 2.6 (SD 2.9) respectively. The proportion of nets that were ITNs was estimated to be around 83% (95% CI: 82-85).

### Bed net use

Sixty-one per cent (95% CI: 57-65) of the 5,151 children under five years of age who were analysed slept under a bed net and 49% (95% CI: 46-52) slept under an ITN. This translates into 80% of the children who slept under a bed net did so under an ITN (95% CI: 78-81).

A total of 3,823 (74.2%) children lived in households that owned at least one bed net and were included in the analysis of determinants of bed net use. Of children who lived in households that owned at least one net 77 % (95% CI: 73-81) slept under a net (any type) the night before the survey and 73% (95% CI: 69-76) did so under an ITN. Eighty one per cent (95% CI: 78-83) of the children living in households who owned at least one ITN slept under an ITN. Table 3 summarizes the association between sleeping under a bed net (any type) the night before of the survey and potential determinants.

**Table 3:** Univariate and multivariable analysis of determinants of bed net use in children < 5 years old living in households owning at least one net

	Total^1,2^		Univariate analysis		Multivariable Analysis^3^	
		n(%)	OR (95%CI)	**p-value**^**4**^	OR (95%CI)	**p-value**^**4**^
**Sex**						
Male	1896	1445(76.21)	1.00			
Female	1927	1489(77.27)	1.06(0.90-1.24)	0.45		
**Age (years)**						
< 1	795	656(82.52)	1.00		1.00	
1	800	643(80.38)	0.87(0.71-1.06)		0.82(0.64-1.04)	
2	770	568(73.77)	0.60(0.46-0.77)	< 0.001	0.57(0.44-0.75)	< 0.001
3	768	556(72.40)	0.56(0.44-0.71)		0.50(0.39-0.65)	
4	690	511(74.06)	0.60(0.44-0.84)		0.56(0.41-0.76)	
**Socio-economic Status**						
1^st ^quintile	670	498(74.33)	1.00			
2^nd ^quintile	760	588(77.37)	1.18(0.86-1.61)			
3^rd ^quintile	790	626(79.24)	1.32(0.87-2.01)	0.45		
4^th ^quintile	817	635(77.72)	1.21(0.82-1.78)			
5^th ^quintile	786	587(74.68)	1.01(0.71-1.46)			
**Area**						
Urban	1793	1456(81.20)	1.00		1.00	
Rural	2030	1478(72.81)	0.62(0.42-0.91)	0.018	0.32(0.28-0.68)	0.001
**Year**						
2008	2248	1863(82.87)	1.00		1.00	
2009	1575	1071(68.00)	0.44(0.33-0.58)	< 0.001	0.44(0.32-0.56)	< 0.001
**Sick in last 14 days**						
No	3266	2487(76.15)	1.00		1.00	
Yes	549	440(80.15)	1.26(0.98-1.61)	0.061	1.32(1.03-1.69)	0.030
**Malaria transmitted by mosquitos^6^**						
No	498	412(82.73)	1.00			
Yes	2815	2157(76.63)	0.68(0.43-1.08)	0.097		
**Malaria prevented by bednets^6^**						
No	679	478(70.40)	1.00			
Yes	3144	2456(78.12)	1.50(1.12-2.00)	0.009		
**Is fever a symptom of malaria?^6^**						
No	671	516(76.90)	1.00			
Yes	3148	2414(76.68)	0.99(0.76-1.29)	0.96		
**House Sprayed last year?**						
No	660	494(74.85)	1.00			
Yes	2860	2214(77.41)	1.15(0.87-1.52)	0.29		
**Type of eaves**						
Not Open	2295	1768(77.04)	1.00			
Open	1526	1164(76.28)	0.96(0.66-1.38)	0.81		
**Age of head of household**						
< 30	938	730(77.83)	1.00			
30-44	1614	1264(78.31)	1.02(0.75-1.41)	0.13		
> 44	1189	877(73.76)	0.80(0.54-1.18)			
**Education of head of household**						
None	156	97(62.18)	1.00		1.00	
1-4	353	272(77.05)	2.04(1.28-3.26)	0.043	1.58(1.00-2.50)	0.09
5-9	2139	1642(76.76)	2.01(1.17-3.45)		1.70(0.99-2.91)	
> 9	1070	839(78.41)	2.21(1.15-4.26)		1.58(0.86-2.92)	
**At least one ITN in the household**						
No	782	398(50.90)	1.00		1.00	
Yes	3041	2536(83.39)	4.85(3.61-6.51)	< 0.001	**2008^5^: **3.37(2.20-5.19)	< 0.001
					**2009^5^: **6.64(4.03-10.9)	< 0.001

Crude Odds Ratios (OR) and adjusted OR of the effect of each variable on the likelihood of sleeping under a bed net the night before of the survey (n = 3823).

^1^Only children under 5 from households which owned at least one net were included.

^2^For the purpose of this analysis, missing data in any of the variables analysed were excluded.

^3^Adjusted Odds Ratios and p values using a multiple regression model adjusting for all variables presented in the model.

^4^P values obtained through Wald Tests.

^5^Adjusted Odds Ratios using a model adjusting for all the variables of the model and the interaction of this variable with "year". The p-value for the test of interaction of "year" with "owning at least one ITN" was 0.02

^6^Knowledge questions asked to the household interviewee.

After retaining the six explanatory variables that showed association to the outcome variable in the univariate analysis, the year 2009 was strongly associated with a lower bed net use by children under 5 (OR = 0.42, 95% CI 0.32-0.56 p-value < 0.001). The household owning at least one ITN (as opposed to any untreated net) was associated with net use in the multivariable analysis, and there was evidence that this effect differed between 2008 and 2009 (p-value for test for interaction = 0.02). We therefore present the odds ratio of this variable separately for the two years (OR: 3.37, 95% CI: 2.50-5.19 and OR: 6.64, 95% CI: 4.03-10.94 for 2008 and 2009 respectively). There was no evidence of interaction between the other variables of the model and the year of the survey. A child in the household having been sick during the 14 days prior to the survey, the age of the child and whether the household was in an urban or rural area were strongly associated with bed net use. Education of head of household was weakly associated with bed net use.

### Bed net ownership

**Figure 2:**
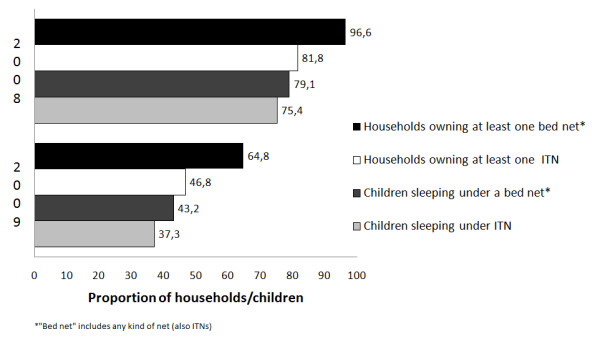
Proportion of households which own at least one bed net or ITN and proportion of children sleeping under a bed net* and ITN per year of survey.

The proportion of households (with at least one child under years of age) owning at least one bed net regardless of whether it was an ITN or not dropped from 97% (95% CI: 95-98) in 2008 to 65% (95% CI: 54-75) in 2009, representing a 32% decrease in eighteen months. ITN ownership declined from 82% (95% CI: 79-84) to 47% (95% CI: 38-55) in the same period (Figure 2). Of the households that owned at least one net of any kind, 84% (95% CI:79-89) in 2008 and 72 (67-78%) in 2009 owned at least one ITN. Since almost all households owned at least one bed net in 2008, only households surveyed in 2009 in the analysis of net ownership were included.

A total of 1693 households with children under five were surveyed during 2009. The proportion of households from urban areas was higher in 2009 (58% in 2009 and 42% in 2008) and the proportion of wealthier households was higher in 2009 (36% in 2008 and 64% in 2009). The other household socio-demographic characteristics in 2009 did not differ from households in 2008 (table 2).

After adjusting for the other variables included in the model, five variables remained independently associated with bed net ownership (Table 4). Having knowledge of malaria transmission and prevention was associated with higher bed net ownership (OR = 1.75, 95% CI: 1.01-3.02 and OR = 1.89, 95% CI: 1.44-2.47 respectively), as well as having the house sprayed in the year prior to the survey (OR = 1.46, 95% CI: 1.05-2.02). A higher number of children (under five) sick in the previous 14 days was associated with higher bed net ownership (p-value of test of trend = 0.007). The type of area (rural or urban) was not associated with owning a bed net.

**Table 4:** Univariate and multivariable analysis of determinants of bed net ownership in households with children < 5 years old

		Houses with at least 1 bed net				
			Univariate analysis		**Multivariable Analysis**^**2**^	
	Total	n(%)	OR (95%CI)	**p-value**^**3**^	OR (95%CI)	**p-value**^**3**^
**Socio-economic Status**						
1st quintile	281	154(54.8%)	1.00			
2nd quintile	327	207(63.3%)	1.42(1.05-1.92)			
3rd quintile	312	216(69.23%)	1.86(1.06-3.26)	0.098		
4th quintile	337	236(70.03%)	1.93(1.12-3.32)			
5th quintile	398	260(65.33%)	1.55(0.78-3.11)			
**Area**						
Urban	954	664(69.6%)	1.00			
Rural	701	409(58.35%)	0.61(0.27-1.37)	0.21		
**Children sick in last 14 days**						
None	1120	676(60.09%)	1.00		1.00	
1	340	256(73.35%)	1.83(1.32-2.53)	0.001	1.66(1.23-2.24)	0.007
2 or more	186	144(77.42%)	2.28(1.53-3.39)		1.94(1.24-3.02)	
**Malaria transmitted by mosquitos^4^**						
No	74	38(51.35%)	1.00		1.00	
Yes	1305	860(65.90%)	1.83(1.19-2.83)	0.01	1.75(1.01-3.02)	0.046
**Malaria prevented by bednets^4^**						
No	382	211(55.24%)	1.00		1.00	
Yes	1273	862(67.71%)	1.70(1.39-2.07)	< 0.001	1.89(1.44-2.47)	< 0.001
**Is fever a symptom of malaria?^4^**						
No	363	235(64.74%)	1.00			
Yes	1288	836(64.91%)	1.01(0.73-1.39)	0.96		
**House Sprayed last year?**						
No	319	186(58.31%)	1.00		1.00	
Yes	1115	768(68.88%)	1.58(1.11-2.25)	0.01	1.46(1.05-2.02)	0.026
**Age of head of household**						
< 30	399	252(63.16%)	1.00			
30-44	717	467(65.13%)	1.09(0.81-1.46)	0.77		
> 44	469	309(65.88%)	1.13(0.79-1.61)			
**Education of head of household**						
none	118	73(61.86%)	1.00			
1-4	87	52(59.77%)	0.92(0.52-1.60)	0.32		
5-9	849	551(64.9%)	1.14(0.76-1.71)			
> 9	529	352(66.54%)	1.23(0.83-1.81)			
**Anyone Pregant last year**						
No	1276	810(63.48%)	1.00			
Yes	379	263(69.39%)	1.30(0.94-1.81)	0.10		
**Been here last 12 months**						
No	236	135(57.20%)	1.00			
Yes	1411	934(66.19%)	1.46(0.93-2.30)	0.09		
**Would you have ACT free?**						
No	479	311(64.93%)	1.00			
Yes	847	544(64.23%)	0.97(0.78-1.21)	0.78		
Don't know	320	211(65.94%)	1.05(0.80-1.37)			
**Head of household is male?**						
No	980	654(66.73%)	1.00			
Yes	605	374(61.82%)	0.81(0.62-1.05)	0.11		
**Number of children under 5**						
1	946	632(66.81%)	1.00		1.00	
2 or more	709	441(62.20%)	0.82(0.61-1.09)	0.16	0.73(0.52-1.03)	0.067

Year 2009 (n = 1655)^1^

^1^For the purpose of this analysis, missing data in any of the variables analysed were excluded.

^2^Adjusted Odds Ratios and p values using a multiple regression model adjusting for all variables present in the model.

^3^P values obtained through Wald Tests.

^4^Knowledge questions asked to the household interviewee.

## Discussion

### Bed net use

The most striking factor associated with bed net utilization was the decline in use from 2008 to 2009. It does not seem that seasonality (surveys were conducted at different times of the year) could explain these findings, since malaria transmission is stable in Bioko throughout the year [30]. However, this lower use of bed nets in 2009 coincided with a marked decrease in ownership (the proportion of households which had at least one ITN dropped more than 35%, which made it, together with the decline reported in Sierra Leona from 2007 to 2008 [1], one of the highest ITN ownership drops in two annual consecutive surveys found in the literature). The reasons for this decline remain unclear. There is speculation that some nets were taken to be given away or sold in the continental region of Equatorial Guinea, where people from Bioko had relatives and where there were two provinces that did not benefit from a mass distribution campaign. Nets may also have been discarded because they were physically damaged [31] (through wear and tear or partially eaten by rats), or because people thought that they would receive new replacement nets free-of-charge again through a similar mass campaign (households were not counselled on the need to retain their nets as long as possible given that no mass resupply was planned).

Apart from ownership, five other variables were independently associated with children under five using a bed net. The strongest association was with household ownership of ITN as opposed to any net. Households with untreated nets only were less likely to use them. These households with untreated nets were households that were not reached by the LLIN mass distribution, or, for reasons not yet understood, disposed of the nets between the delivery and the survey. It could also be that untreated nets were purchased for decorative or comfort purposes for adults' beds, so children sleeping apart were less likely to benefit from them. Even though the current recommendation of WHO is to use LLINs, untreated nets have also been shown to reduce the incidence of uncomplicated and severe malaria cases [5].

In common with other studies in African countries [18], the older the child, the less likely he/she was to sleep under a bed net the night prior to the survey. When there are only a limited number of bed nets, it could be that priority is given to the younger children, or perhaps, as the children grow older and experience fewer episodes of malaria, there may be a false sense of protection, leading to lower utilization of bed nets. There was also good evidence suggesting that if a child was sick at some point in the 14 days prior to the survey they were more likely to sleep under a bed net. This suggests that an increased awareness of risk brought about by being sick may translate into better compliance with malaria protection. This finding suggests the need for education as a means of achieving increased and appropriate bed net utilization. However, the hypothesis that children living in higher risk areas are more likely to use a net (reverse causality) cannot be excluded.

The type of setting was also an important factor predicting bed net use. Children from rural areas were less likely to sleep under a bed net, compared with their urban counterparts. This information is highly relevant, since many malaria cases occur in rural areas. An association between area and bed net ownership was not found, suggesting a difference of attitude or lack of knowledge in rural areas, rather than a problem of access to nets. This could mean that IEC campaigns were more successful in promoting net use in urban areas such as Malabo.

SES appeared to be unrelated to the likelihood of bed net use or ownserhip, as already described for other countries by Goesch *et al *and Chase *et al *[13,17,32], supporting the idea that the effect of SES might be country-specific. Since LLINs were distributed free of charge in Bioko, it was less likely that net ownership (and in consequence net use), would depend on household wealth.

### Bed net ownership

Bioko Island, as the rest of Equatorial Guinea, has been undergoing an economic boom associated with the discovery and exploitation of oil and gas reserves. This economic growth has resulted in a substantial growth in housing as living standards have improved. The BIMCP's IRS program has found that there has been average annual growth in houses of 20% between 2004 and 2010. It is possible that an increase in housing could be associated with an escalation in sleeping areas as less congested housing meant individuals did not need to share sleeping areas. The growth in housing and sleeping areas, coupled with population growth [estimated annual population growth rate (2000-2008): 2.8%][33], has meant that the LLINs distributed in 2007 were inadequate to meet the needs of the population in 2008 and 2009. Therefore, the decline in ownership may have been partially explained by increased housing and sleeping areas in addition to population growth, and nets being given away, sold or discarded due to dilapidation. The fact that there have been no other important sources of treated bed nets in the country since 2007 (for new families and replacement of damaged nets) is probably a major reason for the sharp decline in ownership.

Occupants of houses that were sprayed in the previous 12 months were more likely to own at least one bed net. These households could have been more sensitive to dangers of malaria, suggesting therefore both owning a net and having a house recently sprayed would be signs of enhanced awareness. Another explanation could also be that households sprayed in the previous 12 months were less remote and perhaps households with access to IRS are more likely to have access to other interventions, such as ITN delivery.

Bed net ownership was directly associated with answering correctly two of the three questions assessing knowledge in the multivariable analysis. It seems reasonable that if caregivers knew how malaria was transmitted and prevented, they were more likely to protect their children with nets. This supports the notion as reported elsewhere [34,35] that health education yields changes in health seeking behaviour related to malaria prevention. Paradoxically, knowing whether fever was a symptom of malaria was not associated with higher ownership. Since this symptom has high sensitivity but low specificity for diagnosing malaria, it is plausible that this finding resulted from the fact that fever could be perceived as a symptom of many diseases rather than a complete lack of knowledge about malaria symptoms.

A surprising finding was that the greater the number of children under 5 in the household, the less likely it was that the household owned a bed net. It could have been that new households formed after the mass distribution in 2007 were more likely to have young children. This result needs further investigation since the under-five age group is of particular concern, even if the policy is one of universal coverage. Another unexpected finding was the association of being sick in the last 14 days and higher ownership. It could be that those children are perhaps sick more often and households retain their bed nets for longer periods, or it could be that they try to buy bed nets from the scarce sell points in the island, but this argument is thought to be unlikely

The findings of this study must be viewed with some caution due to a number of limitations in the analytic methodology employed. First, as with all studies based on cross-sectional survey data assessments of causality are problematic and, therefore, all the conclusions presented here are what it is believed are the most plausible hypothesis to explain the results. Reverse causality is likely in some of the findings. For example in the association between ownership of nets and children being sick in the last 14 days, the sickness episodes could be a result of not being protected by a net because the household did not own one.

Second, Principal Component Analysis was used to create a score as a proxy for socio-economic status. The extent to which this score represents the true socio economic status of a household was not validated for this particular setting. The variables included were a subjective choice by the authors based on previous literature of SES indexes, and therefore the final scores depend on the variables selected. As some authors already described [29], infrastructure variables could have introduced geographical bias since more people with high socio economic status live in urban areas, but this might also have been an accurate reflection of the distribution of household wealth.

Finally, it is possible that there was recall bias, especially with the questions regarding household sprayed in the last year, being sick in the last 14 days, education of head of household (in case the interviewee was not the head of household), and even whether the individual slept under a net the night before the survey.

## Conclusions

Bed net ownership was by far the factor most strongly associated with use in Bioko Island. The sharp decline in ownership from 2008 to 2009 was a striking finding of this study. If not reversed, this could undermine some of the progress previously made, as for example the two-thirds reduction in child mortality [3]. There is an urgent need to identify the reasons for the decline in net ownership that has been observed in Bioko and elsewhere following mass distributions. This will help providers to put in place more effective measures by which coverage can be increased through more regular distribution of nets and routine access to replacement nets. Subsequent surveys in Bioko include questions to respondents of households that have had nets but no longer have any to assess the reasons why nets are lost or discarded. IEC promotion of use of nets should include messages about care and maintenance of nets and the expected lifespan of an LLIN. Knowledge of the usefulness of bed nets in malaria prevention was found to be important especially for bed net ownership. The utilization of bed nets was lower in rural areas even though ITN ownership was similar in urban and rural areas. This suggests that there is a need to enhance the promotion of the use of nets in rural areas through targeted IEC. Finally, misperceptions that older children are less at risk of contracting malaria, or only using nets once a child has been sick, should also be specifically addressed within IEC messages.

## Competing interests

The funders had no role in study design, data collection and analysis, decision to publish, or preparation of the manuscript. Marathon Oil and other members of the funding consortium have no commercial interest in the findings made in this article.

## Authors' contributions

ALGB developed the study protocol, participated in the design and analysis, and drafted the manuscript. AR participated in the analysis of the paper and commented on the paper. IK supervised and conceived of the study and helped in drafting the manuscript. CS and GN coordinate the BIMCP and revised the manuscript. AM helped in the acquisition of surveys and drafted the manuscript. All authors read and approved the final manuscript.
